# Reply to: “Management of transient ischemic attack in low- and middle-income country: a critical analysis”

**DOI:** 10.1055/s-0046-1822632

**Published:** 2026-07-16

**Authors:** Samia Talise El Horr de Moraes, Maramelia Miranda-Alves, Leticia Rebello, Sheila O. Martins, Fabricio Oliveira Lima, Wagner Mauad Avelar, Rodrigo Bazan, Marcos C. Lange

**Affiliations:** 1 Universidade Federal do Paraná, Hospital de Clínicas, Curitiba PR, Brazil.; 2 Universidade Federal de São Paulo, Escola Paulista de Medicina, Hospital São Paulo, São Paulo SP, Brazil.; 3 Hospital de Base de Brasília, Brasília DF, Brazil.; 4 Universidade Federal do Rio Grande do Sul, Hospital de Clínicas de Porto Alegre, Porto Alegre RS, Brazil.; 5 Hospital Moinhos de Vento, Porto Alegre RS, Brazil.; 6 Hospital Geral de Fortaleza, Fortaleza CE, Brazil.; 7 Universidade Estadual de Campinas, Campinas SP, Brazil.; 8 Universidade Estadual Paulista, Faculdade de Medicina de Botucatu, Botucatu SP, Brazil.

Dear Editor,

We would like to thank Varão and Nogueira for their careful reading of our article, “Transient Ischemic Attack in the Practice of Neurology in a Low- and Middle-Income Country”, and for their constructive comments, which provide an opportunity to clarify specific aspects of our findings.

We agree that the management of transient ischemic attack (TIA) in low- and middle-income countries must be interpreted within the context of structural limitations, including restricted access to advanced neuroimaging, heterogeneous availability of stroke units, and limited implementation of specialized TIA clinics. These factors were central to the rationale and interpretation of our survey and were deliberately emphasized to avoid direct extrapolation from high-income healthcare settings.

Regarding the apparent discrepancy in the reported percentages related to the duration of antiplatelet therapy, we acknowledge that this resulted from a data reporting error. The percentage corresponding to the 14 respondents who reported maintaining antiplatelet therapy for up to 21 days was incorrectly reported as 56%; the correct value is 11%, based on the appropriate denominator. We regret this error and appreciate the opportunity to correct it. This correction does not affect the overall interpretation of the results, which continue to demonstrate substantial variability in antiplatelet therapy duration and reflect the lack of consensus in clinical practice. As discussed in the manuscript, the high proportion of missing or non-applicable responses to treatment-related questions remains an important methodological limitation and warrants cautious interpretation of subgroup analyses. Future surveys would benefit from clearer denominator stratification and enhanced data validation, particularly for clinically relevant variables such as dual antiplatelet therapy duration.

With respect to the impact of the coronavirus disease 2019 (COVID-19) pandemic, this variable was included for descriptive purposes, aiming to capture whether respondents perceived disruptions in TIA and stroke care during that period. Given the exploratory nature of the survey and the breadth of variables assessed, a detailed analytical evaluation of the specific effects of the pandemic was beyond the scope of the study.

Finally, we concur with the authors' caution regarding the high non-response rate to key management questions, such as anticoagulation in cardioembolic TIA. This limitation was explicitly acknowledged in our manuscript and highlights the challenges inherent in capturing real-world clinical practice through voluntary surveys. Rather than diminishing the relevance of the findings, this observation underscores the need for standardized protocols, structured continuing education initiatives, and more robust methodological approaches in future studies.

In conclusion, we appreciate the opportunity to address these points. We believe that the issues raised reinforce the main conclusions of our study, namely that TIA management in Brazil is characterized by significant variability influenced by systemic constraints, and that targeted educational and organizational strategies are necessary to improve adherence to evidence-based care in low- and middle-income countries.

